# Sodium oxybate for the maintenance of abstinence in alcohol-dependent patients: An international, multicenter, randomized, double-blind, placebo-controlled trial

**DOI:** 10.1177/02698811221104063

**Published:** 2022-07-07

**Authors:** Julien Guiraud, Giovanni Addolorato, Mariangela Antonelli, Henri-Jean Aubin, Andrea de Bejczy, Amine Benyamina, Roberto Cacciaglia, Fabio Caputo, Maurice Dematteis, Anna Ferrulli, Anna E Goudriaan, Antoni Gual, Otto-Michael Lesch, Icro Maremmani, Antonio Mirijello, David J Nutt, François Paille, Pascal Perney, Roch Poulnais, Quentin Raffaillac, Jürgen Rehm, Benjamin Rolland, Claudia Rotondo, Bruno Scherrer, Nicolas Simon, Katrin Skala, Bo Söderpalm, Lorenzo Somaini, Wolfgang H Sommer, Rainer Spanagel, Gabriele A Vassallo, Henriette Walter, Wim van den Brink

**Affiliations:** 1 Department of Psychiatry, Amsterdam Neuroscience, Amsterdam UMC, University of Amsterdam, Amsterdam, The Netherlands; 2 D&A Pharma, Paris, France; 3 Alcohol Use Disorder and Alcohol Related Disease Unit, Department of Internal Medicine and Gastroenterology, Fondazione Policlinico Universitario A. Gemelli IRCCS, Rome, Italy; 4 Internal Medicine Unit, Columbus-Gemelli Hospital, Department of Internal Medicine and Gastroenterology, Fondazione Policlinico Universitario A. Gemelli IRCCS, Rome, Italy; 5 French Institute of Health and Medical Research (Inserm), Centre de Recherche en Epidémiologie et Santé des Populations (CESP), Universite Paris-Saclay, Villejuif, France; 6 Addiction Research and Treatment Center, Paul Brousse Hospital, Paris-Sud University, Villejuif, France; 7 Section of Psychiatry and Neurochemistry, Institute of Neuroscience and Physiology, Sahlgrenska Academy, University of Gothenburg, Goteborg, Sweden; 8 Laboratorio Farmaceutico CT, San Remo, Italy; 9 Department of Internal Medicine, SS. Annunziata Hospital, Cento (Ferrara), University of Ferrara, Italy; 10 Centre for the Study and Treatment of Alcohol-Related Diseases, Department of Translational Medicine, University of Ferrara, Ferrara, Italy; 11 Department of Addiction Medicine, Grenoble-Alpes University Hospital, and Faculty of Medicine, Grenoble Alpes University, France; 12 Department of Endocrinology, Nutrition and Metabolic Diseases, IRCCS MultiMedica, Milan, Italy; 13 Department of Biomedical Sciences for Health, University of Milan, Milan, Italy; 14 Department of Psychiatry, Amsterdam University Medical Centers, University of Amsterdam, Amsterdam, The Netherlands; 15 Arkin, Department of Research and Quality of Care, Amsterdam Public Health Research Institute, Amsterdam, The Netherlands; 16 Psychiatry Department, Neurosciences Institute, Hospital Clinic, IDIBAPS, Barcelona, Spain; 17 University Clinic of Psychiatry and Psychotherapy, Department of Social Psychiatry, Medical University of Vienna, Austria; 18 Santa Chiara University Hospital, University of Pisa, Italy; 19 Department of Medical Sciences, IRCCS Casa Sollievo della Sofferenza General Hospital, San Giovanni Rotondo (FG), Italy; 20 Centre for Neuropsychopharmacology, Imperial College London, United Kingdom; 21 Department of Addiction Treatment, University Hospital, Vandoeuvre-lès-Nancy, France; 22 Addiction Medicine, CHU Nîmes, France; 23 Institute for Mental Health Policy Research, Centre for Addiction and Mental Health, Toronto, Ontario, Canada; 24 Department of Psychiatry, Dalla Lana School of Public Health, University of Toronto, Toronto, Ontario, Canada; 25 Clinical Psychology & Psychotherapy Technical University Dresden, Dresden, Germany; 26 Department of International Health Projects, Institute for Leadership and Health Management, I.M. Sechenov First Moscow State Medical University, Moscow, Russia; 27 SUAL, HCL, CH Le Vinatier; Univ Lyon; UCBL; INSERM U1028; CNRS UMR5292, Centre de Recherche en Neuroscience de Lyon (CRNL), Bron, France; 28 Centro di Riferimento Alcologico della Regione Lazio (CRARL), Dipartimento di Salute Mentale, Roma, Italy; 29 Bruno Scherrer Conseil, Saint Arnoult en Yvelines, France; 30 Aix Marseille Univ, APHM, INSERM, IRD, SESSTIM, Hop Sainte Marguerite, Department of Clinical Pharmacology, CAP-TV, Marseille, France; 31 Department of Child and Adolescent Psychiatry, Medical University of Vienna, Austria; 32 Addiction Treatment Center, Local Health Unit, ASL Biella, Italy; 33 Medical Faculty, Institute of Psychopharmacology, Central Institute of Mental Health, University of Heidelberg, Mannheim, Germany; 34 Institute of Psychopharmacology, Central Institute of Mental Health, Heidelberg University, Mannheim, Germany; 35 Department of Internal Medicine, Barone Lombardo Hospital, Canicattì, Italy

**Keywords:** Alcohol dependence, alcohol use disorders, maintenance of abstinence, sodium oxybate, GHB, RCT

## Abstract

**Background:**

Sodium oxybate (SMO) has been shown to be effective in the maintenance of abstinence (MoA) in alcohol-dependent patients in a series of small randomized controlled trials (RCTs). These results needed to be confirmed by a large trial investigating the treatment effect and its sustainability after medication discontinuation.

**Aims:**

To confirm the SMO effect on (sustained) MoA in detoxified alcohol-dependent patients.

**Methods:**

Large double-blind, randomized, placebo-controlled trial in detoxified adult alcohol-dependent outpatients (80% men) from 11 sites in four European countries. Patients were randomized to 6 months SMO (3.3–3.9 g/day) or placebo followed by a 6-month medication-free period. Primary outcome was the cumulative abstinence duration (CAD) during the 6-month treatment period defined as the number of days with no alcohol use. Secondary outcomes included CAD during the 12-month study period.

**Results:**

Of the 314 alcohol-dependent patients randomized, 154 received SMO and 160 received placebo. Based on the pre-specified fixed-effect two-way analysis of variance including the treatment-by-site interaction, SMO showed efficacy in CAD during the 6-month treatment period: mean difference +43.1 days, 95% confidence interval (17.6–68.5; *p* = 0.001). Since significant heterogeneity of effect across sites and unequal sample sizes among sites (*n* = 3–66) were identified, a site-level random meta-analysis was performed with results supporting the pre-specified analysis: mean difference +32.4 days, *p* = 0.014. The SMO effect was sustained during the medication-free follow-up period. SMO was well-tolerated.

**Conclusions:**

Results of this large RCT in alcohol-dependent patients demonstrated a significant and clinically relevant sustained effect of SMO on CAD.

**Trial registration:**

ClinicalTrials.gov Identifier: NCT04648423

## Introduction

Alcohol dependence (AD; World Health Organization, 2016) occurs in 2.6% of people aged 15+ years worldwide (World Health Organization, 2018) and can result in a reduction of life expectancy by up to 35 years as compared with the general population (Rehm et al., 2018).

One of the treatment goals for AD is abstinence (European Medicines Agency, 2010). Currently, disulfiram, acamprosate, and naltrexone are registered for the maintenance of abstinence (MoA) in AD patients. Although effective on the group level, effects sizes are limited, and many AD patients fail to respond to these medications (European Medicines Agency, 2010; van den Brink et al., 2018). Therefore, additional pharmacological treatments are needed.

Sodium oxybate (SMO), as an oral solution, has been approved in Italy and Austria for the treatment of alcohol withdrawal syndrome and the MoA since 1991 and 1999, respectively (van den Brink et al., 2018). SMO is the sodium salt of γ-hydroxybutyric acid (GHB), a short-chain fatty acid that is naturally synthesized in the mammalian brain. GHB is a gamma-aminobutyric acid (GABA) receptor agonist which binds with low affinity to GABA subtype B receptors (and indirectly with the GABA subtype A receptors) and with high affinity to GHB-specific receptors (Keating, 2014). Given that the pharmacological profile of GHB has some similarities to that of alcohol, one proposed mechanism of SMO in the treatment of AD is its ability to mimic some effects of alcohol in the brain particularly to reduce craving while abstinent (Kamal et al., 2016; Keating, 2014). SMO 50 mg/kg/day showed the evidence of efficacy compared with placebo or naltrexone in the MoA in AD patients in a series of open label and blinded randomized controlled trials (RCTs) and was positively evaluated for this indication in a Cochrane review (Caputo et al., 2003, 2007; Gallimberti et al., 1992; Leone et al., 2010). However, studies were generally small with sample sizes ranging from 16 to 86 patients and they did not investigate the sustainability of the SMO effect after treatment discontinuation.

The present RCT (GATE 2) in 314 AD patients aimed to confirm the efficacy and safety of oral SMO in the MoA. Secondary aims included the assessment of sustained SMO effects during the 6-month medication-free period immediately following the 6-month treatment period and monitoring the risk of SMO dependence.

## Methods

### Design

This double-blind, placebo-controlled, outpatient RCT with balanced randomization (1:1) included patients from 11 sites in Austria, Germany, Italy, and Poland. The trial was conducted in accordance with the ethical principles of the Declaration of Helsinki, Good Clinical Practices, and the European guidelines for the development of AD treatment (Plinius Maior Society, 1994). The study was approved by ethics committees/institutional review boards at all sites and written informed consent was obtained from all the patients. The trial is registered in ClinicalTrials.gov (NCT04648423).

In a previous review by Skala et al. (2014) on SMO in the treatment of AD, some preliminary information on the GATE 2 trial was provided. The detailed study protocol is provided in Supplement 2.

### Participants

Inclusion criteria were as follows: age 21–75 years, a clinical diagnosis of Diagnostic and Statistical Manual of Mental Disorders, 4th Edition (DSM-IV) and International Classification of Diseases, 10th Revision (ICD-10) AD based on an AD checklist, a Cutting down, Annoyance by criticism, Guilty feeling, and Eye-openers (CAGE; Ewing, 1984) score ⩾ 2, a Munich Alcoholism Test (MALT) (Feuerlein et al., 1979) score ⩾ 11, availability of a responsible relative or caregiver, and a successful detoxification, including a 10-day treatment period and a subsequent 10-day untreated abstinent period. Exclusion criteria were as follows: relapse during the detoxification period; renal failure, severe respiratory problems, heart failure; hepatic encephalopathy stage II-IV; drug dependence; history of epilepsy or epileptic seizures not properly controlled by established anti-epileptic treatment; severe psychiatric disorder requiring medical treatment; treatment with clonidine, disulfiram (after the end of the detoxification period), haloperidol, bromocryptine, serotonine re-uptake inhibitors, or other serotoninergic agents; female subjects who cannot assure not to become pregnant during the study; and pre-existent hypersensitivity to GHB.

### Treatments/interventions

The statistical department of the clinical research organization involved in the study established the allocation sequence. The randomization was stratified by site and the random numbers were computer generated using a pseudo-random uniform distribution with a block size of four patients to ensure a good balance of treatment groups within the sites. The study medications (SMO and placebo) were supplied by the sponsor of the study and packed in identical bottles of 140 ml, numbered according to the allocation sequence. The investigators assigned the eligible subjects to interventions using the lowest unassigned number available in the site. Sponsor, investigators, and patients were blind to treatment assignment during the full study period. Blinding was not broken for any patient during the trial. SMO (175 mg/ml) and placebo oral solutions were identical in appearance and taste.

### Procedures

Randomized patients entered a 6-month treatment phase with SMO or placebo followed by an abrupt discontinuation of the study medication and a 6-month medication-free period. Patients self-administered the medication at the dose of 17.5 ml/day in three doses for patients with a bodyweight ⩽65 kg and 20 ml/day in three doses for others. In an amendment, these doses were increased to 19 ml/day for patients ⩽65 kg and 22.5 ml for others to be closer to the approved posology in Italy and Austria (50 mg/kg/day). Out of 314 randomized patients, the original and the revised dose regimen were received by 11 and 303 patients, respectively. Standard psychosocial interventions at the individual sites were provided at each visit to enhance motivation and abstinence from alcohol. Study visits were planned for every month in the treatment phase and every 2 months during the follow-up phase. Patients received a diary card to record drinking and non-drinking days.

### Measures

Baseline data included the following: date of birth, gender, race, height, body weight, ICD-10 AD diagnosis, DSM IV AD diagnosis, CAGE score, MALT score, mean corpuscular volume (MCV), and γ-glutamyl transferase (GGT).

The primary efficacy outcome was the cumulative abstinence duration (CAD) during the 6-month treatment phase. CAD was the primary endpoint recommended in the Plinius Maior Society guidelines for the evaluation of treatments of AD (Plinius Maior Society, 1994). European guidelines have since then evolved from 2010 onwards and the proportion of patients continuously abstinent throughout the treatment period (continuous abstinence rate (CAR)) is now the recommended primary endpoint for studies on MoA (European Medicines Agency, 2010). However, at the time the GATE 2 study was designed (2000), CAD was still considered the standard primary outcome for studies on the treatment of AD. For example, CAD was widely utilized as the (co-)primary endpoint in acamprosate trials, including those that were used as pivotal evidence in the registration process of the drug for MoA in the European Union (Spanagel and Mann, 2005). Consequently, it was also defined as a primary outcome in the Cochrane meta-analysis of acamprosate for the MoA in AD patients (Rösner et al., 2010a). CAD is still considered an important secondary endpoint by the European Medicines Agency (2010). In the current study, CAD was calculated as the number of days with no alcohol use (Plinius Maior Society, 1994). At treatment group level, CAD measures the differences in CAR as well as the differences in abstinence duration in relapsing patients. It can therefore be conceptualized as a composite endpoint with the current recommended primary endpoint as one of its components. In GATE 2 and due to uncertainty regarding accurate reporting of duration of relapses, if a relapse occurred since the last visit and was reported by the patient at a visit, the entire month before the visit was considered as a period of relapse, irrespective of the declared duration of the relapse (Besson et al., 1998; Gual and Lehert, 2001; Pelc et al., 1997; Plinius Maior Society, 1994; Poldrugo, 1997; Tempesta et al., 2000; Whitworth et al., 1996). Relapse was defined as any alcohol consumption.

Key secondary outcome measures include the following: the CAD during the 12-month study period, the CAR at the end of the 6-month treatment phase and at the end of the 12-month observation period, the time to first relapse, the MCV and GGT at the end of 6-month treatment, and the compliance with the assigned treatment. CAR definition was compliant with the definition of the European guidelines (European Medicines Agency, 2010). Compliance with assigned treatment was defined as sufficient if the total actual consumption of the medication was higher than 75% of the total intended consumption.

Main safety assessments included the evaluation of Adverse Events (AEs) and the Lubeck Craving Recurrence Risk questionnaire (Veltrup, 1994, items 1 and 2) to evaluate craving for the study medication. Patients were asked to define the frequency of their desire for the study medication using the following categories: (1) (nearly) continuously from getting up in the morning until going to sleep; (2) approximately every 15–30 min; (3) approximately every 30–60 min; (4) every 2–3 hours; (5) more seldom than every 2–3 hours; and (6) never.

### Statistical methods

The sample size calculation was based on a group difference between placebo and SMO of 20 days of CAD during the treatment period and a standard deviation (SD) of 60 days. Using the assumed variability and a two-sided α = 0.05, 143 patients in each treatment group would provide a power of 80%. Given the randomization procedure with block size of four patients and to reduce the risk of having a site with no patient in one treatment group, it was decided to increase the sample size to up to 160 patients per group.

All analyses were conducted in the Intent-to-Treat (ITT) population which includes all patients who received at least one dose of the allocated drug.

CAD was analyzed in accordance with the pre-specified analysis in the protocol, including a fixed-effect two-way analysis of variance (ANOVA) with terms for treatment, site, and treatment-by-site interaction. Heterogeneity of effect across sites was first identified by graphical display of the results for each individual site. Consequently, to explore the generalizability of results and to substantiate the robustness of the point estimate of the treatment effect, mixed-effect models with treatment as fixed effect and site and site-by-treatment interaction as random effects were fitted to the data (Barr et al., 2013; Feaster et al., 2011; Senn, 2021). Unfortunately, these models faced convergence issues in the estimation of the variance of the random terms. This commonly occurs with small-to-medium data sets and/or in complex models with several terms and/or with models including a categorical variable (such as site) as random effect and with a relatively small number of categories (Barr et al., 2013; Bates et al., 2015, 2018; Eager and Roy, 2017). In this context and as an alternative method to the mixed-effect models, site-level random-effect meta-analyses were fitted to the data for both CAD at the end of 6-month treatment and CAD at the end of the 12-month study period. Treatment effects were computed at site level and were then pooled using a random-effect meta-analysis model. Heterogeneity was tested with the Cochran Q test and was quantified with the I^2^ index. The relationship between treatment effect and placebo response in CAD in each site was post-hoc investigated with a linear regression model.

CAR was analyzed using risk difference with 95% confidence intervals (CIs). Time to the first relapse during the treatment period was analyzed with the Kaplan–-Meier estimates. MCV and GGT were summarized with descriptive statistics (geometric mean). Mean difference in the compliance with the assigned treatment was tested with a Student’s t-test. The effect of the site on the treatment effect was a posteriori investigated with a two-way ANOVA with site-by-treatment interaction for compliance as outcome and with a site-level meta-analysis for CAR as outcome.

Dropout and missing data were assumed to be missing not at random and were considered as relapse to alcohol for CAD, CAR, and time to first relapse. This assumption was selected because relapse was the main documented reason for dropout in previous trials (Balldin et al., 2003; Geerlings et al., 1997; Paille et al., 1995; Pelc et al., 1997; Poldrugo, 1997; Sass et al., 1996; Wiesbeck, 2001). MCV and GGT at the end of treatment as well as compliance with assigned treatment were analyzed based on observed values. A sensitivity analysis was conducted on the primary endpoint with missing data assumed to be missing at random and using multiple imputation.

All AEs were coded according to the Medical Dictionary for Regulatory Activities dictionary. The proportions of patients that reported AEs were tabulated by group and compared by means of the Chi-square or Fisher’s exact probability test. For additional information on the above analyses, see Supplements 1 and 2.

The principal statistical software used was SAS^®^, Version 9.4. PROC MIXED was used for performing fixed-effect ANOVA and mixed-effects models as well as site-level random-effect meta-analysis on the primary endpoint.

## Results

**Figure 1.**
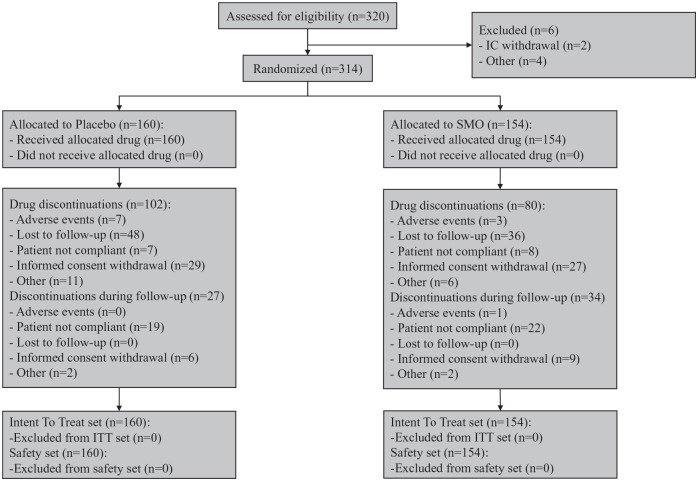
Patient flow chart.

From July 2001 to March 2011, 320 subjects were screened and 314 participants were included in the ITT population, 154 were randomized to receive SMO and 160 to receive placebo. A total of 182 of the 314 randomized patients (58.0%) did not complete the 6-month treatment phase. Non-completion rates were lower in the SMO than in the placebo group both at the end of treatment (52% vs 64%) and at the end of study period (74% vs 81%) (Figure 1).

There were no clinically relevant differences in baseline demographic or clinical characteristics between the two groups (Table 1).

**Table 1.** Demographics and baseline clinical characteristics: mean (SD).

Characteristics	SMO	Placebo
*N*	154	160
Age (years)	44.3 (8.7)	44.5 (9.8)
Gender: females, *n* (%)	33 (21.4)	31 (19.4)
Race, *n* (%)		
White	150 (97.4)	158 (98.8)
Other	4 (2.6)	2 (1.2)
Height (cm)	172.4 (8.7)	174.0 (7.7)
Body mass index (kg/m^2^)	25.3 (3.7)	25.4 (4.1)
AD diagnosis		
ICD-10^a^	5.3 (1.0)	5.4 (0.9)
DSM-IV^a^	6.1 (1.3)	6.3 (1.1)
CAGE score	3.5 (0.7)	3.5 (0.6)
MALT 1 score	2.0 (3.0)	2.4 (3.0)
MALT 2 score	19.2 (2.9)	19.2 (3.3)
MALT 1+2 score	21.2 (4.5)	21.5 (4.6)
Mean corpuscular volume (fL)^b^	94.4	94.6
GGT (U/L)^b^	46.5	43.9

MALT 1 evaluates the presence of polyneuropathy, delirium tremens, and/or liver disease with four points score per each positive answer; MALT 2 evaluates 24 items with one point score per each positive answer.

a Number of AD diagnosis criteria met.

b Geometric mean.

AD, alcohol dependence; GGT, γ-glutamyl transferase; CAGE, Cutting down, Annoyance by criticism, Guilty feeling, and Eye-openers; MALT, Munich Alcoholism Test; MCV, mean corpuscular volume.

### Primary endpoint

**Figure 2.**
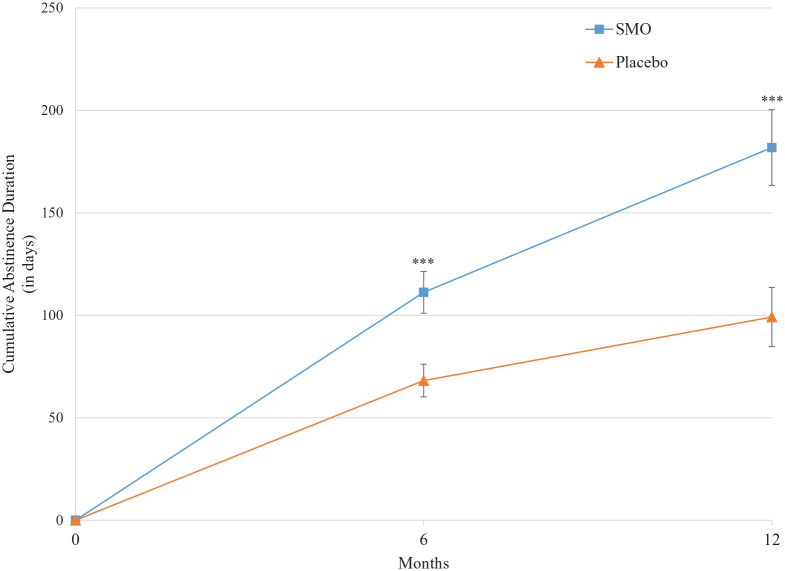
Adjusted mean CAD over the study period. Bars indicate standard error; ***: *p* ⩽ 0.001. CAD, cumulative abstinence duration.

The adjusted mean CAD during the 6-month treatment period was significantly higher in the SMO group than in placebo arm in both the fixed-effect model (adjusted mean difference +43.05 days, *p* = 0.001) and the site-level random-effect meta-analysis (mean difference +32.37 days, *p* = 0.014) (Figure 2 and Table 2).

**Table 2.** CAD during the 6-month treatment period and during the 12-month study period.

In days	SMO (*N* = 154) Adj. mean (SE)	Placebo (*N* = 160) Adj. mean (SE)	Adj. mean difference (95% CI)	*p* Value
CAD during the 6-month treatment period				
Fixed-effect model				
Pre-specified analysis	111.20 (10.19)	68.15 (7.95)	43.05 (17.61–68.49)	0.001
Sensitivity analysis	148.20 (9.53)	120.65 (8.33)	27.55 (2.47–52.63)	0.032
Random-effect meta-analysis	NA	NA	32.37 (6.45–58.28)	0.014
CAD during the 12-month study period				
Fixed-effect model	181.84 (18.50)	99.19 (14.44)	82.65 (36.47–128.83)	<0.001
Random-effect meta-analysis	NA	NA	58.04 (8.54–107.53)	0.022

adj., adjusted; CAD, cumulative abstinence duration; CI, confidence interval; NA, not available; SE, standard error.

Results of the sensitivity analysis with multiple imputation supported the pre-specified analysis (fixed-effect model: adjusted mean difference +27.55 days, *p* = 0.032). Due to a negative estimated τ^2^, it was not possible to provide multiple imputation results for the site-level random-effect meta-analysis.

**Figure 3.**
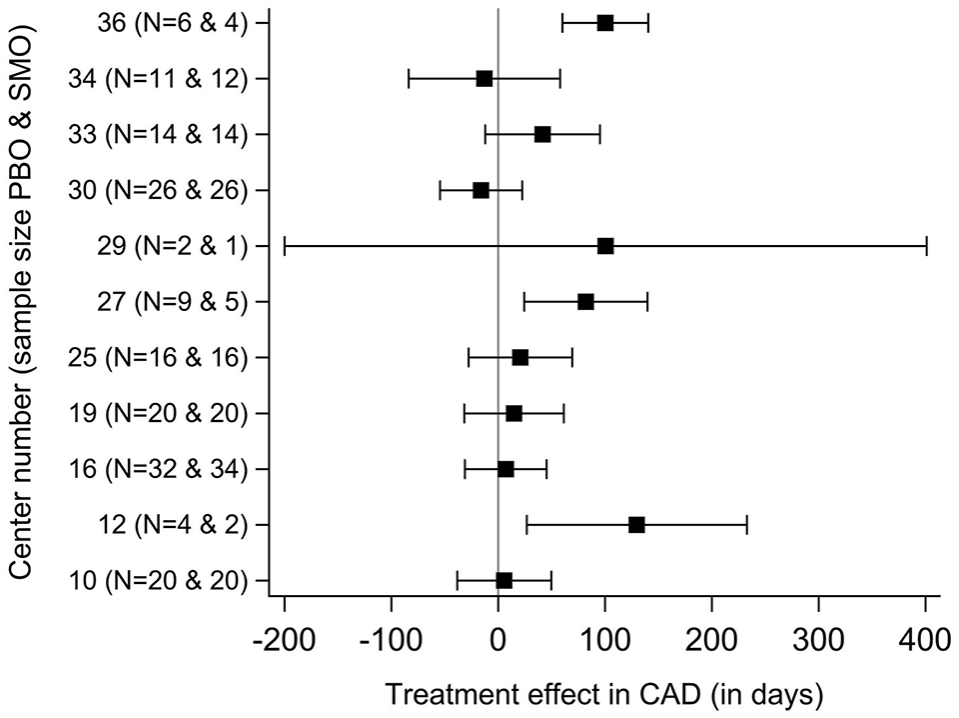
Site-specific treatment effects (95% CI) in CAD during the 6-month treatment period. CAD, cumulative abstinence duration; CI, confidence interval; PBO: placebo; SMO: sodium oxybate.

The site fixed effect on the CAD was not significant (*p* = 0.40), but a potential treatment-by-site interaction was identified (*p* = 0.16). Interestingly, in the meta-analysis model, the Cochran Q test was highly significant (*p* = 0.001) and substantial heterogeneity of the treatment effect across sites was identified (*I*^2^ = 60.8%, 95% CI: 24.2–79.7%; Figure 3).

**Figure 4.**
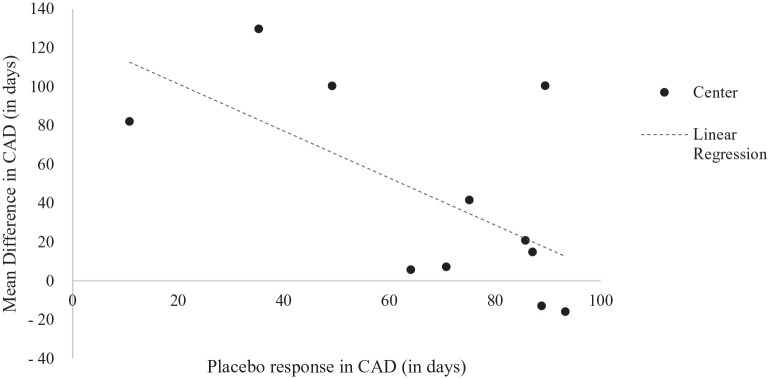
Mean difference in CAD during the 6-month treatment period and mean placebo response in each site. CAD, cumulative abstinence duration.

The estimated treatment effect across sites varied from −16 days to +130 days of CAD and was negatively correlated (*r* = −0.63; *p* = 0.04) with the placebo response in the sites (Figure 4). The treatment effect was numerically in favor of SMO in 9 of the 11 sites (Figure 4) and significantly in favor of SMO in two sites (Supplemental Table S2).

### Secondary endpoints

The adjusted mean CAD at the end of the 12-month observation period was in favor of SMO: adjusted mean group difference +82.65 days (*p* < 0.001) in the fixed-effect model and mean group difference +58.04 days (*p* = 0.022) in the random-effect meta-analysis model (Table 2; Figure 2).

The CAR was 25.3% in SMO group and 20.0% in placebo group (*p* = 0.25) at the end of the 6-month treatment period and 15.6% in SMO group compared to 10.6% in placebo group (*p* = 0.19) at the end of the observation period (Supplemental Tables S3 and S4). The random-effect meta-analysis of CAR provided similar results. The median time to first relapse during the treatment period was 77 days in the SMO group compared to 46 days in the placebo arm (difference + 31 days; *p* = 0.13).

Regarding MCV and GGT, values at the end of treatment were similar in both treatment groups and improved similarly in both treatment groups compared with screening: mean GGT of 33.4 U/L at day 180 (vs 46.5 U/L at screening) in SMO group and 30.6 U/L at day 180 (vs 43.9 U/L at screening) in placebo group; mean MCV of 91.0 fL at day 180 (vs 94.4 fL at screening) in SMO group and 92.0 fL at day 180 (vs 94.6 fL at screening) in placebo group.

Compliance was high in both groups and the mean difference was not significantly different: mean (SD) of 93.5% (14.9) in the SMO group and of 91.4% (14.5) in the placebo group (*p* = 0.21). When site and site-by-treatment interaction were included in the model, the point estimate for compliance and the *p* value was improved in favor of SMO but results did not reach statistical significance.

### Safety

The most frequently reported AEs were dizziness and nausea with similar incidence rates in the two groups (Table 3). The number of patients with AEs leading to discontinuation of study medication was lower in SMO group (six patients) than in the placebo group (11 patients). The most experienced AE leading to discontinuation was nausea with two (1.3%) patients in the SMO group and dizziness with three (1.9%) patients in the placebo group.

**Table 3.** Treatment emergent adverse events: safety population.

Preferred term	SMO (*n* = 154)	PBO (*n* = 160)	*p* value
AE	29 (18.8)	32 (20.0)	0.79
AE reported by at least two patients			
Dizziness	9 (5.8)	8 (5.0)	0.74
Nausea	4 (2.6)	5 (3.1)	0.78
Headache	3 (1.9)	3 (1.9)	0.96
Vomiting	3 (1.9)	2 (1.3)	0.62
Bronchitis	2 (1.3)	0 (0.0)	0.29
Arthralgia	0 (0.0)	2 (1.3)	0.31
Disturbance in attention	0 (0.0)	2 (1.3)	0.31
Somnolence	2 (1.3)	0 (0.0)	0.29
Alcohol withdrawal syndrome	1^a^ (0.6)	2^a^ (1.3)	0.58
Alcoholism	0 (0.0)	3^b^ (1.9)	0.21
Delirium tremens	2^c^ (1.3)	0 (0.0)	0.29
Dermatitis	1 (0.6)	1 (0.6)	0.98
SAE	6 (3.9)	6 (3.8)	0.95
AE treatment related	14 (9.1)	11 (6.9)	0.47
SAE treatment related	2 (1.3)	1 (0.6)	0.62
AE leading to discontinuation	6 (3.9)	11 (6.9)	0.24
Fatal AE	1 (0.6)	0 (0.0)	0.49

Data are numbers of patients (%).

*p* Value based on Chi-square test except for SAE treatment related and fatal AE (Fisher’s exact test).

a These events occurred during the treatment period: one was considered serious (placebo group), one related to study medication (placebo group), and one not serious and not related to study medication (SMO group).

b Craving for or relapse to alcohol (with hospitalization in one case).

c None were considered to be serious or to be related to study medication. These events occurred during the follow-up (e.g., untreated) period (one at day 224 and one at day 291).

AE, adverse events; PBO, placebo; SAE, serious adverse events; SMO, sodium oxybate.

One death was reported in the SMO group: the patient was murdered while consuming alcohol. Five patients in the SMO group experienced non-fatal serious AEs (SAEs) compared with six patients in the placebo group. A total of three patients experienced SAEs that were considered by the investigator to be related to study medication: one overdose and one suicidal depression (SMO), one drug toxicity (placebo).

No AE related to abuse or misuse were reported. The craving for medication was similar in both treatment groups at day 180 (SMO group: mean (standard error) 38.21 (2.93), placebo group: 37.98 (3.40) on a scale of 1–100) and remained of the same magnitude at follow-up visits without any significant difference between treatment groups. At day 180, 98.6% of patients in the SMO and 96.6% of patients in the placebo group reported having no desire to take study medication or a desire to take study medication more seldom than every 2–3 h in the last 30 days. At follow-up visits, these proportions remained of the same magnitude as for day 180 without any significant difference between treatment groups.

## Discussion

SMO has previously shown efficacy in the MoA in short-term RCTs (Caputo et al., 2003, 2007; Gallimberti et al., 1992; Guiraud et al., 2021; Leone et al., 2010; van den Brink et al., 2018). The current double-blind placebo-controlled RCT confirmed these findings showing a statistically significant and clinically relevant effect of SMO in the pre-specified fixed-effect model of the primary endpoint, CAD during 6-month treatment with a mean difference of +43 days. In addition, the effect of SMO in terms of CAD was still present at the end of the 12-month observation period.

The estimated treatment effect across sites varied from −16 days to +130 days of CAD and a potential site-by-treatment interaction was identified, suggesting heterogeneity of treatment effect. To provide a statistical basis for the generalization of the intervention results to the total AD population from which the sites were randomly selected, site-level random-effect meta-analyses were applied. Results showed point estimates of the treatment effect consistent with those from the fixed-effect two-way ANOVA and indicated an important heterogeneity of treatment effect across sites.

Heterogeneity of the SMO effect in the MoA has also been observed in previous SMO RCTs with a larger effect size in patient populations with a lower placebo response rate (Guiraud et al., 2021; van den Brink et al., 2018). This heterogeneity in efficacy is not specific to the treatment of AD with SMO. In a meta-analysis of 51 RCTs for AD, the variability of the effect sizes of acamprosate and naltrexone across trials was substantial and the treatment effect estimates were significantly negatively correlated with the placebo response in the study population (Litten et al., 2013). In the current trial, the placebo response in terms of CAD (mean 73 days at study level) was higher than expected (40–50 days) and the treatment effect was negatively correlated with the placebo response at site level: the lower the placebo response, the higher the treatment effect in the site. Although this post-hoc finding should be interpreted with caution, it is important to further study moderators of SMO treatment effect and the predictors of the placebo response. For example, recent subgroup analyses of RCTs and a meta-regression of 19 RCTs found higher placebo responses in AD patients with more than 14 consecutive days of abstinence prior to randomization (Gueorguieva et al., 2011, 2012; Scherrer et al., 2021; van den Brink et al., 2018). In the GATE 2 study, only patients with a detoxification period of at least 20 days were included and this may explain the relatively high placebo response at study level. There is a convergence of evidence that the duration of abstinence before treatment initiation and/or the baseline alcohol consumption could be moderators of the effect of SMO in AD (Guiraud et al., 2021; Scherrer et al., 2021; van den Brink et al., 2018). Unfortunately, these baseline data were not collected in the current study. We are aware that also other subgroupings, for example, according to genetic, neurobiological, and other clinical features, might be important as predictors for the SMO treatment effect. They represent decisive factors for course, therapy, and outcome (Lesch et al., 2020). Interestingly, SMO has previously shown efficacy with large effect sizes in treatment-resistant AD patients (Maremmani et al., 2001) and also in RCTs conducted in high-severity population, that is, in patient populations with a low response rate to placebo (van den Brink et al., 2018). Consequently, in Italy, SMO was approved for the MoA in treatment-resistant AD patients only.

The current study also showed a sustained effect of SMO on CAD 6 months after the study medication discontinuation. The treatment effect in CAD was higher at the end of the study period than at the end of the treatment period and was clinically relevant. The duration of the follow-up period in trials in the treatment of AD is still debated among the scientific community and regulatory agencies. Based on data indicating that abstinence at 6 months has been shown to be a predictor of long-term abstinence, the US Food and Drug Administration (2015) does not require any specified follow-up period in confirmatory trials for AD. On the other hand, some researchers considered that post-treatment evaluations had to include at least 12 weeks of observation (Rösner et al., 2010b), whereas the European Medicines Agency recommends a follow-up of 12–15 months (European Medicines Agency, 2010).

CAD is no longer the primary endpoint recommended by European guidelines for studies on MoA. However, CAD measures the differences in CAR, the current primary endpoint recommended by European guidelines. In GATE 2, the statistically significant beneficial effect of SMO in CAD is explained by a numerically higher CAR and a longer abstinence duration in relapsing patients.

The dropout rates in the current study were high but consistent with those commonly observed in AD trials and those from RCTs that were used to establish efficacy of approved compounds in the treatment of AD (European Medicines Agency, 2012; Nice, 2011). In addition, dropouts were considered as drinking days/failures in the CAD and the CAR. Moreover, a sensitivity analysis on the primary endpoint using multiple imputation and a fixed-effect model supported the results of the pre-specified analysis of the primary endpoint. Unfortunately, the estimated τ^2^ was negative in the site-level random-effect meta-analysis, indicating that this sensitivity analysis was not possible with this type of analysis and this data set.

No difference between treatment groups was found in GGT and MCV at the end of treatment. However, GGT and MCV values were almost normal at baseline, possibly due to the long detoxification period (20 days), which left limited room for improvement during the treatment phase.

The 11 study sites were opened almost on a sequential basis with a mean recruitment duration of 1.5 years/site, explaining the recruitment duration of 10 years. However, randomization was stratified by site and the sponsor, investigators, and patients remained blind for the treatment allocation during the full 12-month study period and unblinding took place only after the last patients of the last site completed the study. Therefore, we believe that neither the external nor the internal validity of the study was jeopardized. Only six patients were assessed for eligibility and excluded from the study. This is mainly explained by the fact that the GATE 2 study was conducted concomitantly and at the same sites as the GATE 1 RCT, which tested the equivalence of SMO and oxazepam for treating the alcohol withdrawal syndrome and in which 454 subjects were screened and 128 were randomized (Caputo et al., 2014). As they were fulfilling GATE 2 inclusion criteria, participants who were successfully detoxified with either SMO or oxazepam and who completed the 20-day study period in the GATE 1 trial were invited to participate in the GATE 2 study. Since patients and investigators remained blind to treatment assignment during the study period in both GATE 1 and GATE 2, we do not expect any serious risk of bias in the GATE 2 findings resulting from the recruitment of patients detoxified with SMO. In addition and since criteria for participation were more stringent in GATE 1, patients who were fulfilling GATE 2 inclusion criteria but who were excluded from the GATE 1 study, for instance due to the lack of moderate or severe alcohol withdrawal syndrome, were also invited to participate in the GATE 2 study.

The AE profile was as expected from previously published data from pharmacovigilance and clinical studies (Addolorato et al., 2020) and reflects the pharmacological profile of SMO. No significant group differences were found in the incidence of AEs. The most reported AEs were effects on the nervous system (dizziness) and gastrointestinal apparatus (nausea). No difference in craving for study medication was detected between treatment groups, suggesting a low risk of abuse and dependence to SMO in the study population. One death (murdered) was reported but was not considered to be related to the study medication. Overall, SMO was well-tolerated.

In conclusion, SMO showed efficacy in CAD during the 6-month treatment period in this double-blind RCT. The current RCT confirms efficacy and safety of SMO in the treatment of AD reported in previous RCTs and pharmacovigilance database, especially for patient populations with a low placebo response rate. In this subgroup of severe AD patients, additional data are warranted to further support the clinically relevant effect of SMO.

## Supplemental Material

sj-doc-1-jop-10.1177_02698811221104063 – Supplemental material for Sodium oxybate for the maintenance of abstinence in alcohol-dependent patients: An international, multicenter, randomized, double-blind, placebo-controlled trial Click here for additional data file. Supplemental material, sj-doc-1-jop-10.1177_02698811221104063 for Sodium oxybate for the maintenance of abstinence in alcohol-dependent patients: An international, multicenter, randomized, double-blind, placebo-controlled trial by Julien Guiraud, Giovanni Addolorato, Mariangela Antonelli, Henri-Jean Aubin, Andrea de Bejczy, Amine Benyamina, Roberto Cacciaglia, Fabio Caputo, Maurice Dematteis, Anna Ferrulli, Anna E Goudriaan, Antoni Gual, Otto-Michael Lesch, Icro Maremmani, Antonio Mirijello, David J Nutt, François Paille, Pascal Perney, Roch Poulnais, Quentin Raffaillac, Jürgen Rehm, Benjamin Rolland, Claudia Rotondo, Bruno Scherrer, Nicolas Simon, Katrin Skala, Bo Söderpalm, Lorenzo Somaini, Wolfgang H Sommer, Rainer Spanagel, Gabriele A Vassallo, Henriette Walter and Wim van den Brink in Journal of Psychopharmacology

sj-rar-2-jop-10.1177_02698811221104063 – Supplemental material for Sodium oxybate for the maintenance of abstinence in alcohol-dependent patients: An international, multicenter, randomized, double-blind, placebo-controlled trial Click here for additional data file. Supplemental material, sj-rar-2-jop-10.1177_02698811221104063 for Sodium oxybate for the maintenance of abstinence in alcohol-dependent patients: An international, multicenter, randomized, double-blind, placebo-controlled trial by Julien Guiraud, Giovanni Addolorato, Mariangela Antonelli, Henri-Jean Aubin, Andrea de Bejczy, Amine Benyamina, Roberto Cacciaglia, Fabio Caputo, Maurice Dematteis, Anna Ferrulli, Anna E Goudriaan, Antoni Gual, Otto-Michael Lesch, Icro Maremmani, Antonio Mirijello, David J Nutt, François Paille, Pascal Perney, Roch Poulnais, Quentin Raffaillac, Jürgen Rehm, Benjamin Rolland, Claudia Rotondo, Bruno Scherrer, Nicolas Simon, Katrin Skala, Bo Söderpalm, Lorenzo Somaini, Wolfgang H Sommer, Rainer Spanagel, Gabriele A Vassallo, Henriette Walter and Wim van den Brink in Journal of Psychopharmacology
