# Supplementary material for: Sodium oxybate for the maintenance of abstinence in alcohol-dependent patients: An international, multicenter, randomized, double-blind, placebo-controlled trial
Source: J Psychopharmacol. 2022 Jul 7;36(10):1136–45. doi: 10.1177/02698811221104063 (PMC9548946; doi:10.1177/02698811221104063)
Supplement: sj-doc-1-jop-10.1177_02698811221104063 – Supplemental material for Sodium oxybate for the maintenance of abstinence in alcohol-dependent patients: An international, multicenter, randomized, double-blind, placebo-controlled trial [file sj-doc-1-jop-10.1177_02698811221104063.doc]

**Supplementary Online Content 1**

Table of content

[1. Inclusion/exclusion criteria 2](#__RefHeading___Toc84438126)

[**1.1. List of inclusion criteria** 2](#__RefHeading___Toc84438127)

[**1.2. List of exclusion criteria** 2](#__RefHeading___Toc84438128)

[**1.3. MALT** 3](#__RefHeading___Toc84438129)

[2. Primary endpoint definition 3](#__RefHeading___Toc84438130)

[**2.1. Primary endpoint definition** 3](#__RefHeading___Toc84438131)

[**2.2. Sensitivity analyses** 4](#__RefHeading___Toc84438132)

[3. Secondary endpoints definition 4](#__RefHeading___Toc84438133)

[4. Results 6](#__RefHeading___Toc84438134)

[**4.1. Patients enrolled in each country** 6](#__RefHeading___Toc84438135)

[**4.2. Treatment effect for primary endpoint in each center** 6](#__RefHeading___Toc84438285)

[4.3. Efficacy results for Continuous Abstinence Rate (CAR) 7](#__RefHeading___Toc84438435)

1. Inclusion/exclusion criteria
   1. List of inclusion criteria

The following inclusion criteria were applied:

- males and females;
- of any ethnic group;
- age between 21 and 75 years at recruitment;
- clinical diagnosis of DSM-IV and ICD-10 alcohol dependence (AD) based on an AD checklist;
- a CAGE score ≥ 2 and a Munich Alcoholism Test (MALT) score ≥ 11 further supporting the DMS-IV and ICD-10 AD diagnosis
- classified according to Lesch;
- having successfully undergone a detoxification program, encompassing a 10-day treatment period and a subsequent 10-day untreated follow-up;
- with a responsible relative or caregiver;
- having issued the informed consent.
  1. List of exclusion criteria

The following exclusion criteria were applied:

- subjects who did not quit alcohol drinking after the detoxification period;
- subjects with history of epilepsy or epileptics seizures not properly controlled by established anti-epileptic treatment;
- subjects with dependence from narcotics or other drugs of abuse;
- subjects without a stable address;
- subjects without a reference relative or caregiver;
- subjects with renal failure (blood creatinine >2.5 mg/dl and/or documented proteinuria >500 mg/day);
- subjects with heart failure or severe respiratory failure;
- subjects with hepatic encephalopathy stage II-IV;
- subjects with severe psychiatric disorders requiring treatment with psychoactive medications (excluding short-term benzodiazepine treatments);
- subjects under treatment with clonidine, disulfiram (after the end of the detoxification period), haloperidol, bromocryptine, serotonine re-uptake inhibitors or other serotoninergic agents;
- female subjects who cannot assure not to become pregnant during the 7-month period covering treatment and the first treatment-free month of follow-up;
- documented pre-existent hypersensitivity to GHB;
- subjects unable or unwilling to issue the informed consent;
- participating to another clinical investigation in the previous month prior to recruitment;
- any other medical condition which, according to the investigator, justifies the patient’s exclusion from the study.
  1. MALT

The Munich Alcoholism Test (MAT) consists of two complementary parts: a 7-item physician's assessment part and a 24-item self-assessment part.

In the present study, the physician’s assessment part (MALT 1) was reduced to 3 items (polyneuropathy, delirium tremens, liver disease) with four points score per each positive answer.

The self-assessment part (MALT 2) evaluated the following 24 items with one point score per each positive answer:

1. Recently my hand trembled often
2. On the morning I feel nausea at times
3. Sometimes I tried to get rid of trembling and of nausea with alcohol
4. Currently I feel sad because of my problems and my difficulties
5. It is not unusual that I drink alcohol before lunch
6. After one or two drinks I feel a strong desire to drink more
7. I think much of alcohol
8. Sometimes I have drunk alcohol even against my physician's opinion
9. When I drink much alcohol I tend to eat not much
10. At work I have been criticised because of my drinking
11. I prefer to drink alone
12. Since I started drinking, I am in bad shape
13. I feel often guilty because of drinking
14. I tried to limit drinking to some occasions or some hours of the day
15. I think I should drink less
16. Without alcohol I would have less problems
17. When I am disturbed I drink alcohol to calm down
18. I think that alcohol is destroying my life
19. Sometimes I would like to give up drinking, sometimes not
20. Other people do not understand why I drink
21. I would be better with my spouse if I did not drink
22. Sometimes I tried to go on without alcohol
23. I would be glad if I would not drink
24. Often some people told me that my breath smelled of alcohol
25. Primary endpoint definition
    1. Primary endpoint definition

The primary efficacy outcome was the Cumulative Abstinence Duration (CAD) observed during the 6-month treatment period. CAD was defined as the number of days of abstinence during the observed period.

According to the Statistical Analysis Plan (SAP), CAD was calculated as follows:

*CAD=Days of exposure to treatment period - Sum of drinking days*

In the above reported equation, the number of “Days of exposure to treatment period” was calculated as follows:

*[Date of visit Day 180 (or the previous last visit date available) - Date of visit Day 0]+1*

The number of drinking days was estimated according to the answer to the question “Has the patient been abstinent since last visit?” reported on e-CRF, in the following way:

- if the answer to the question was NO, the number of drinking days was calculated as the days between current and previous visit
- if the answer to the question was YES, the number of drinking days was considered equal to zero.

For patients who dropped out, days between the last study visit attended by the patient and the end of treatment (Day 180) were considered as non-abstinent.

The maximum possible CAD was 181 days, as, according to the protocol, day 0 is included in the calculation if it was an abstinent day.

- 1. Sensitivity analyses

A sensitivity analysis using multiple imputation was conducted on the primary endpoint.

Since the pattern of missingness in CAD was monotone, we applied a monotone data imputation using a predictive mean matching method with treatment and site as predictors. A total of 100 imputed datasets were generated and treatment effect was estimated from these imputed data.

1. Secondary endpoints definition

Secondary analyses were composed of the following endpoints:

1. Continuous Abstinence Rate (CAR) at end of treatment and at end of observation period: abstinence from any alcohol intake during the full observation period till the end of the 6-month treatment period and till the end of the 6-month follow-up period. Patients who dropped out or not reporting at the planned interviews were considered as treatment failure (relapse to alcohol).
2. CAD during the whole observation period.
3. Corrected cumulative abstinence duration (CCAD) during the treatment period, defined as the ratio between CAD calculated as above indicated, and the total duration of treatment exposure.
4. Time to first relapse: for this outcome, early termination was regarded a treatment failure, except in the case of intercurrent illness or protocol violation. This outcome was analyzed using the Kaplan-Meier technique.
5. Values of %CDT (carbohydrate-deficient transferrin) were planned to be analyzed using an ANCOVA model that included treatment as main effect and baseline %CDT as a covariate. The missing data were allocated by a Last (post-baseline) Observation Carried Forward (LOCF) method. No data was imputed for baseline (Day 0) values. However, post baseline %CDT data were collected for only 27% of randomized patients and only 8.6% of randomized patients had observed %CDT data at end of treatment making any analysis meaningless.
6. Intensity and frequency of craving for alcohol was measures with the LCRR (Lübecker Craving Risiko Rückfall) questionnaire (ITEM 1-3). Item1 was analyzed by visits using an ANCOVA model that included treatment as main effect and baseline (Day 0) Item 1 scores as a covariate. Item2 was summarized by means of absolute numbers and percentages of the different categories for each visit. Item3 was analyzed considering the total score by visits, using an ANCOVA model that included treatment as main effect and baseline (Day 0) Item 3 scores as a covariate. The missing data for Item1 and for total score of Item3 were allocated by a Last Observation Carried Forward (LOCF) method. No data was imputed for baseline (Day 0) values.
7. Time course of γ-GT and of MCV, recorded at entry, after three and six months of treatment, and at the end of the post-treatment follow-up. Values and changes of γ-GT and MCV from screening were summarized with descriptive statistics: geometric mean, first quartile (Q1), median, third quartile (Q3), minimum and maximum were reported.
8. Compliance with the assigned treatment during the treatment period was analyzed by study group with descriptive statistics.
9. Results
   1. Patients enrolled in each country

Table S1. Number of patients enrolled in the ITT population per country

|  | **N Screened** | **N Randomized** |
| --- | --- | --- |
| Austria | 41 | 40 |
| Germany | 47 | 46 |
| Italy | 75 | 75 |
| Poland | 157 | 153 |
| Total | 320 | 314 |

- 1. Treatment effect for primary endpoint in each center

**Table S2. Treatment effect in primary endpoint in each site**

| **Site** | **Mean (SD) CAD** | | | |  | **N** | |
| --- | --- | --- | --- | --- | --- | --- | --- |
|  | **SMO** | **PBO** | **Mean Difference** | **p value** |  | **SMO** | **PBO** |
| 10 | 69.70 (68.77) | 64.05 (73.27) | 5.65 | 0.8028 |  | 20 | 20 |
| 12 | 165.00 (11.31) | 35.25 (69.83) | 129.75 | 0.0692 |  | 2 | 4 |
| 16 | 77.88 (75.08) | 70.78 (83.12) | 7.10 | 0.7166 |  | 34 | 32 |
| 19 | 101.95 (80.28) | 87.1 (69.83) | 14.85 | 0.5363 |  | 20 | 20 |
| 25 | 106.50 (68.52) | 85.75 (71.72) | 20.75 | 0.4093 |  | 16 | 16 |
| 27 | 92.80 (88.05) | 10.78 (16.91) | 82.02 | 0.0163 |  | 5 | 9 |
| 29 | 190.00 | 89.50 (125.16) | 100.50 | 0.6306 |  | 1 | 2 |
| 30 | 77.31 (71.52) | 93.31 (70.00) | -16.00 | 0.4188 |  | 26 | 26 |
| 33 | 116.71 (62.44) | 75.14 (81.02) | 41.57 | 0.1404 |  | 14 | 14 |
| 34 | 75.83 (80.28) | 88.82 (93.24) | -12.98 | 0.7233 |  | 12 | 11 |
| 36 | 149.50 (41.68) | 49.17 (24.09) | 100.33 | 0.0012 |  | 4 | 6 |

- 1. Efficacy results for Continuous Abstinence Rate (CAR)

**Table S3. Countinuous Abstinence Rate during the 6-month treatment period**

|  | | | **SMO (N=154)** | **Placebo (N=160)** | **P-value^*^** |
| --- | --- | --- | --- | --- | --- |
| N (%) of patients abstinent at each treatment visit | Day 30 | n (%) | 104 (67.5%) | 89 (55.6%) | 0.030 |
|  | Day 60 | n (%) | 87 (56.5%) | 79 (49.4%) | 0.206 |
|  | Day 90 | n (%) | 73 (47.4%) | 62 (38.8%) | 0.122 |
|  | Day 120 | n (%) | 66 (42.9%) | 54 (33.8%) | 0.097 |
|  | Day 150 | n (%) | 63 (40.9%) | 48 (30.0%) | 0.043 |
|  | Day 180 | n (%) | 57 (37.0%) | 44 (27.5%) | 0.071 |
| N (%) of patients abstinent during 6-month treatment period | | n (%) | 39 (25.3%) | 32 (20.0%) | 0.259 |

^*^ p-value based on Chi-square test.

**Table S4. Continuous Abstinence Rate during the 12-month study period**

|  | | | **SMO (N=154)** | **Placebo (N=160)** | **P-value^*^** |
| --- | --- | --- | --- | --- | --- |
| N (%) of patients abstinent at each follow-up visit | Day 240 | n (%) | 41 (26.6%) | 34 (21.3%) | 0.264 |
|  | Day 300 | n (%) | 34 (22.1%) | 24 (15.0%) | 0.106 |
|  | Day 360 | n (%) | 33 (21.4%) | 23 (14.4%) | 0.103 |
| N (%) of patients abstinent during the 12- month study period | | n (%) | 24 (15.6%) | 17 (10.6%) | 0.192 |

^*^ p-value based on Chi-square test.
